# Correction: Clustering of Vector Control Interventions Has Important Consequences for Their Effectiveness: A Modelling Study

**DOI:** 10.1371/journal.pone.0101625

**Published:** 2014-06-27

**Authors:** The PLOS ONE Staff

## Notice of Republication

This article was republished on June 3rd, 2014, due to the figures being ordered incorrectly. The publisher apologizes for this error. Please download the PDF again to view the corrected article. The originally published, uncorrected article and the republished, corrected article are provided here for reference.

## Supporting Information

File S1 Originally published, uncorrected article.
(PDF)

File S2 Republished, corrected article.
(PDF)
