# Supplementary material for: Correction: Clustering of Vector Control Interventions Has Important Consequences for Their Effectiveness: A Modelling Study
Source: PLoS One. 2014 Jun 27;9(6):e101625. doi: 10.1371/journal.pone.0101625 (PMC4074199; doi:10.1371/journal.pone.0101625)
Supplement: File S2 — Republished, corrected article. (PDF) [file pone.0101625.s002.pdf]

OPEN ACCESS Freely available online

PLOS ONE

Clustering of Vector Control Interventions Has Important Consequences for Their Effectiveness: A Modelling Study

Angelina Mageni Lutambi[1,2,3]*, Nakul Chitnis[1,2,4], Olivier J. T. Briët[1,2], Thomas A. Smith[1,2], Melissa A. Penny[1,2]

1 Epidemiology and Public Health, Swiss Tropical and Public Health Institute, Basel, Switzerland, 2 University of Basel, Basel, Switzerland, 3 Ifakara Health Institute, Dar es Salaam, Tanzania, 4 Fogarty International Center, National Institutes of Health, Bethesda, Maryland, United States of America

Abstract

Vector control interventions have resulted in considerable reductions in malaria morbidity and mortality. When universal coverage cannot be achieved for financial or logistical reasons, the spatial arrangement of vector control is potentially important for optimizing benefits. This study investigated the effect of spatial clustering of vector control interventions on reducing the population of biting mosquitoes. A discrete-space continuous-time mathematical model of mosquito population dynamics and dispersal was extended to incorporate vector control interventions of insecticide treated bednets (ITNs), Indoor residual Spraying (IRS), and larviciding. Simulations were run at varying levels of coverage and degree of spatial clustering. At medium to high coverage levels of each of the interventions or in combination was more effective to spatially spread these interventions than to cluster them. Suggesting that when financial resources are limited, unclustered distribution of these interventions is more effective. Although it is often stated that locally high coverage is needed to achieve a community effect of ITNs or IRS, our results suggest that if the coverage of ITNs or IRS are insufficient to achieve universal coverage, and there is no targeting of high risk areas, the overall effects on mosquito densities are much greater if they are distributed in an unclustered way, rather than clustered in specific localities. Also, given that interventions are often delivered preferentially to accessible areas, and are therefore clustered, our model results show this may be inefficient. This study provides evidence that the effectiveness of an intervention can be highly dependent on its spatial distribution. Vector control plans should consider the spatial arrangement of any intervention package to ensure effectiveness is maximized.

Citation: Lutambi AM, Chitnis N, Briët OJT, Smith TA, Penny MA (2014) Clustering of Vector Control Interventions Has Important Consequences for Their Effectiveness: A Modelling Study. PLoS ONE 9(5): e97065. doi:10.1371/journal.pone.0097065

Editor: Joshua Yukich, Tulane University School of Public Health and Tropical Medicine, United States of America

Received August 16, 2013; **Accepted** April 14, 2014; **Published** May 13, 2014

Copyright: © 2014 Lutambi et al. This is an open-access article distributed under the terms of the Creative Commons Attribution License, which permits unrestricted use, distribution, and reproduction in any medium, provided the original author and source are credited.

Funding: Funding support was from the Bill and Melinda Gates Foundation provided through Swiss TPH. The funders had no role in study design, data collection and analysis, decision to publish, or preparation of the manuscript.

Competing Interests: The authors have the following interests: Thomas A Smith is an Academic Editor for PLOS ONE. This does not alter the authors' adherence to all the PLOS ONE policies on sharing data and materials.

* E-mail: angelina-m.lutambi@unibas.ch

Introduction

Efforts to reduce malaria transmission have lead to the development of efficient vector control interventions, particularly insecticide treated nets (ITNs)(which includes conventional nets treated with a WHO recommended insecticide and long-lasting insecticidal nets). [1], indoor residual spraying (IRS), and larviciding [2–6]. These interventions are currently widely used in malaria endemic countries especially those in sub-Saharan Africa [7] and have lead to a substantial reduction in malaria morbidity and mortality. Nevertheless, malaria continues to claim hundreds of thousands of lives every year [7], thus necessitating a continued control effort to fight the disease. While over $2 billion is invested each year in procuring and distributing vector control interventions [8] for malaria control, this funding is insufficient to achieve universal coverage [8] and it is not clear if this will be sustained given current economic constraints.

Mosquito flight from one place to another [9–12] is affected by several factors including wind, odour, blood and nectar sources, availability of breeding sites, mating, and other ecological and environmental factors [13,14]. The probability that a mosquito will encounter areas that are in receipt of a particular vector control intervention while flying is dependent on the spatial arrangement of the intervention. This probability is also dependent on the complexity of how this interacts with patterns of mosquito movement. This means that it is not obvious how this dependence affects the effectiveness of interventions in controlling malaria. An understanding of how spatial clustering of interventions modifies effectiveness is particularly relevant when financial resources are insufficient, or when logistic constraints make it difficult to achieve universal coverage. It has been unclear how to prioritise the spatial allocation of interventions in such situations.

While the World Health Organization (WHO) strategy on vector management provides information on improving the efficacy, cost-effectiveness, ecological soundness and sustainability of vector control [6], there is limited relevant information on the influence of spatial distribution of these interventions on effectiveness. Approaches coupling both theory and empirical evidence are needed to evaluate and measure effectiveness of interventions at different degrees of spatial distribution for each level of intervention coverage. Despite the importance of these approaches, their development and integration in vector control programmes has been receiving inadequate attention.

Mathematical models play an important role in assessing interventions [15]. Many studies evaluate intervention effective-

ness [16–25], depending on intervention coverage [16,22–24] and the significance of distribution of hosts and breeding sites for malaria transmission [20,25]. Some studies consider spatial and network models [19,20,25,26] while others consider spatial distributions of mosquito populations [27,28]. These models allow the evaluation of interventions by coverage or by any combination of intervention packages [19].

In contrast to these studies, this paper focuses on the spatial distribution of interventions rather than on heterogeneity in distribution of hosts and breeding sites. Using insights from a recent study on mosquito movements [29], a spatial model of vector population dynamics and interventions is used to assess the impact of spatial distribution of vector control interventions on reducing the population of biting mosquitoes. The effects are explored at different coverage levels to provide theoretical evidence on the existence of variability in intervention effectiveness, depending on their spatial distribution in small areas like villages.

Methods

A discrete-space continuous-time mathematical model of mosquito population dynamics and dispersal [29] was extended to incorporate ITN, IRS, and larviciding interventions. The model includes six stages of the mosquito life and feeding cycle: three juvenile stages (egg (E), larval (L), pupal (P)) and three adult stages (host seeking (A_h), resting (A_r), and oviposition site searching (A_o)). The population dynamics of mosquitoes in each stage are described by ordinary differential equations. The discrete space used in the model is a grid made up of hexagons called patches that allows any representation of spatial distribution of hosts and breeding sites and mosquito movement (dispersal) between patches. Dispersal of adult mosquitoes searching for hosts or breeding sites is restricted to the nearest six neighbouring patches.

Model Equations with Interventions

As described in more detail in [29], the population dynamics of mosquitoes are governed by the recruitment of new mosquitoes through the average number of eggs laid per oviposition, b, the development/progression rate from one stage to the next, ρ, the stage specific mortality, μ, the movement rates of host seeking, β^H, and oviposition site searching mosquitoes, β^B. The dynamics of each stage of the life cycle in patch (i,j) with interventions and movement are described using ordinary differential equations:

$$\frac{dE_{(i,j)}}{dt} = b_{(i,j)}\rho_{A_o(i,j)}A_{o(i,j)} - \left(\mu_{E(i,j)} + \rho_{E(i,j)}\right)E_{(i,j)},$$

$$\begin{aligned}\frac{dL_{(i,j)}}{dt} = {} & \rho_{E(i,j)}E_{(i,j)} - \left(\mu_{L_1(i,j)} + \rho_{L(i,j)}\right)L_{(i,j)} \\ & - \mu_{L_2(i,j)}L^2_{(i,j)},\end{aligned}$$

$$\frac{dP_{(i,j)}}{dt} = (1-\varepsilon_{\mathrm{LV}})\rho_{L(i,j)}L_{(i,j)} - \left(\mu_{P(i,j)} + \rho_{P(i,j)}\right)P_{(i,j)},$$

$$\begin{aligned}\frac{dA_{h(i,j)}}{dt} = {} & \rho_{P(i,j)}P_{(i,j)} + \rho_{A_o(i,j)}A_{o(i,j)} \\ & - \left(\mu_{A_{h(i,j)}} + \rho_{A_{h(i,j)}}\right)A_{h(i,j)} \\ & - \gamma_{\mathrm{ITN}(i,j)}\mu_{A_{h(i,j)}}A_{h(i,j)} - \Psi^H_{\mathrm{out}}A_{h(i,j)} + \Psi^H_{\mathrm{in}}A_{h_{\xi'}},\end{aligned}$$

$$\begin{aligned}\frac{dA_{r(i,j)}}{dt} = {} & \rho_{A_{h(i,j)}}A_{h(i,j)} - \left(\mu_{A_{r(i,j)}} + \rho_{A_{r(i,j)}}\right)A_{r(i,j)} \\ & - \gamma_{\mathrm{IRS}(i,j)}\mu_{A_{r(i,j)}}A_{r(i,j)},\end{aligned}$$

$$\begin{aligned}\frac{dA_{o(i,j)}}{dt} = {} & \rho_{A_{r(i,j)}}A_{r(i,j)} - \left(\mu_{A_{o(i,j)}} + \rho_{A_{o(i,j)}}\right)A_{o(i,j)} \\ & - \Psi^B_{\mathrm{out}}A_{o(i,j)} + \Psi^B_{\mathrm{in}}A_{o_{\xi'}}.\end{aligned}$$

The terms $\gamma_{\mathrm{ITN}(i,j)}\mu_{A_{h(i,j)}}A_{h(i,j)}$ and $\gamma_{\mathrm{IRS}(i,j)}\mu_{A_{r(i,j)}}A_{r(i,j)}$ are additional mortality terms due to ITNs and IRS respectively. The term $(1-\varepsilon_{\mathrm{LV}})\rho_{L(i,j)}L_{(i,j)}$ represents the reduced number of larvae developing to pupae from untreated breeding sites, where $\varepsilon_{\mathrm{LV}}$ represents the proportion of breeding sites in a given patch covered by larviciding. Parameters $\Psi^H_{out} = \sum_{\xi' \in N(i,j)} \beta^H_{(i,j)/\xi'}$ and $\Psi^H_{in} = \sum_{\xi' \in N(i,j)} \beta^H_{\xi'/(i,j)}$ represent dispersal out and into patch i,j for host seeking adults respectively, and $N(i,j)$ is a set of six nearest neighbours to patch (i,j) and $\xi' \in N(i,j)$ [29]. Similarly, $\Psi^B_{\mathrm{out}} = \sum_{\xi' \in N(i,j)} \beta^B_{(i,j)/\xi'}$ and $\Psi^B_{\mathrm{in}} = \sum_{\xi' \in N(i,j)} \beta^B_{\xi'/(i,j)}$ represent dispersal out and into patch i,j for oviposition site searching adults. Details of calculation of β are provided in [29]. H and B represent hosts and breeding sites respectively. The remaining parameter definitions and their corresponding values are given in Table 1.

Modelling of the Killing Effects of ITNs and IRS

ITNs kill and prevent access to people for host seeking malaria vectors, thus providing personal protection against malaria to the individuals using them [1,30]. ITNs also provide community protection to non-users [31] due to their killing effects which reduce mosquito longevity. Here, ITNs deployed in a patch are assumed to kill mosquitoes directly, hence affecting the density of host seeking adults in that patch. The killing effect of ITNs in the host seeking stage is modelled as additional mortality to normal mortality associated with host seeking process in the absence of ITNs.

IRS is the application of insecticides on the indoor walls and roofs of houses primarily to kill resting adult mosquitoes. IRS reduces malaria transmission by reducing the vector's life span and population density of vectors [32], but provides little direct personal protection against bites. Although some ingredients used in IRS may repel mosquitoes, this study considers only those

Table 1. Parameter definitions and values used in model simulations [29].

Parameter	Description	Units	Baseline	Source
b	number of eggs laid per oviposition	–	100	[53]
ρ_E	egg hatching rate	day^{-1}	0.50	[53], [54],[55]
ρ_L	rate at which larvae develop into pupae	day^{-1}	0.14	[56], [57], [58]
ρ_P	rate at which pupae develop into adults	day^{-1}	0.50	[53],[54]
μ_E	egg mortality rate	day^{-1}	0.56	[59]
μ_{L_1}	density-independent larval mortality rate	day^{-1}	0.44	[59]
μ_{L_2}	density-dependent larval mortality rate	day^{-1} mosq.$^{-1}$	0.05	
μ_P	pupal mortality rate	day^{-1}	0.37	[59]
ρ_{A_h}	rate at which host seeking mosquitoes enter the resting state	day^{-1}	0.46	[29,60]
ρ_{A_r}	rate at which resting mosquitoes enter oviposition site searching state	day^{-1}	0.43	[60]
ρ_{A_o}	oviposition rate	day^{-1}	3.0	[60]
μ_{A_h}	mortality rate of mosquitoes searching for hosts	day^{-1}	0.18	[29,60]
μ_{A_r}	mortality rate of resting mosquitoes	day^{-1}	0.0043	[60]
μ_{A_o}	mortality rate of mosquitoes searching for oviposition sites	day^{-1}	0.41	[60]

doi:10.1371/journal.pone.0097065.t001

without repellency. Therefore, only the direct killing effect to resting adult mosquitoes is considered.

For ITNs, we let γ_{ITN} be the model parameter for additional mortality of host seeking adults and for IRS, we let γ_{IRS} be the model parameter for additional mortality of resting adults. To compare interventions, γ_{ITN} and γ_{IRS} are expressed as functions of intervention efficacy where efficacy is defined as the ability of an intervention to reduce mosquito survival proportionally. For ITNs or IRS, efficacy, ε_{I}, (where I represents ITNs or IRS) is given by

$$\varepsilon_{\mathrm{I}} = \frac{S_0 - S_{\mathrm{I}}}{S_0}. \quad (1)$$

Here S_0 represents the survival probability of mosquitoes in the absence of an intervention in a given mosquito stage given by

$$S_0 = \frac{\rho_s}{\mu_s + \rho_s}, \quad (2)$$

and S_{I} represents the survival probability of mosquitoes in the presence of interventions in a given stage given by

$$S_{\mathrm{I}} = \frac{\rho_s}{\mu_T + \rho_s}. \quad (3)$$

In equations (2) and (3), ρ_s is the development rate of a mosquito from stage s to the next stage, and μ_s (per unit time) is the natural mortality rate of a mosquito in stage s in the absence of an intervention. μ_T (per unit time) is the total mortality rates of mosquitoes in stage s in the presence of interventions expressed by:

$$\mu_T = \mu_s + \mu_s \gamma_{\mathrm{I}}. \quad (4)$$

Here, γ_{I} (unitless) is a multiplicative factor associated with the effect of intervention I (ITN or IRS). The term $\mu_s\gamma_{\mathrm{I}}$ represents additional mortality of intervention, I. In order to obtain the expression for γ_{I}, we substitute equations (2), (3), and (4) into (1) to obtain

$$\gamma_{\mathrm{I}} = \frac{\varepsilon_{\mathrm{I}}(\rho_s + \mu_s)}{\mu_s(1-\varepsilon_{\mathrm{I}})}. \quad (5)$$

Using the stage specific parameter values for ρ_s, and μ_s [29], with $\varepsilon_{\mathrm{I}} \in [0,1]$, the relationship between γ_{I} and ε_{I} is shown in Figure 1. As would be expected model intervention parameters γ_{I}

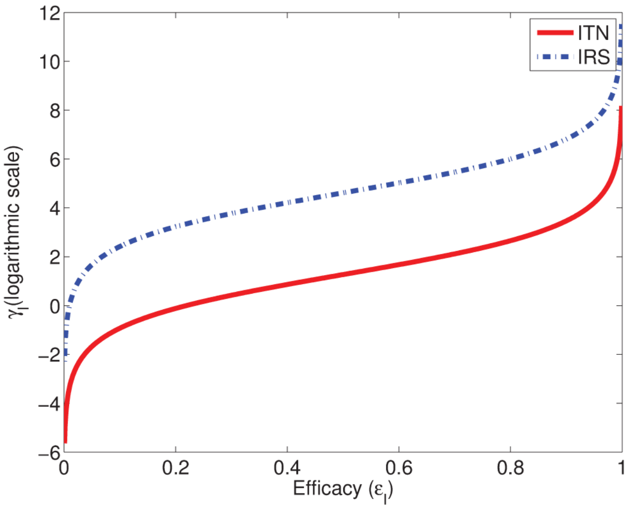

Figure 1. Relationship between ITN and IRS intervention parameters to efficacy (Equation 5 of main text).
doi:10.1371/journal.pone.0097065.g001

increase with increasing efficacy of ITNs or IRS, with IRS showing higher values of γ_I compared to ITNs.

Modelling the Effect of Larviciding

Larviciding is the application of insecticides to mosquito breeding sites targeting the larval stages of the mosquitoes. Studies show that larviciding kills all larvae in treated breeding sites [33–35] and has proved to be important in suppressing the number of malaria transmitting mosquitoes in certain areas [3,33–36]. However, where breeding sites are scattered, field studies show that it is difficult to find and treat the majority of productive breeding sites [37]. The effect of larviciding in the model is to reduce the development of larvae into pupae and thus include a parameter representing the proportion of breeding sites identified and treated within patch (i,j), as $\epsilon_{LV}(i,j)$. The proportion $(1-\epsilon_{LV}(i,j))$ represents the untreated breeding sites, where larvae develop into pupae.

Modelling ITN Repellency

In addition to the killing effect of ITNs that directly affects the density of host seeking adults, the pyrethroid insecticide used to treat nets has a repellent effect acting as a chemical barrier that irritates host seeking mosquitoes as they come close to the nets. Repellency of nets reduces the availability of blood to mosquitoes, increases host searching time, and subsequently prolongs the mosquito gonotrophic cycle duration which in turn impacts mosquito population size. We model the repellent effect of ITNs as follows:

Let Pc be the proportion of hosts within a patch who are covered by ITNs (patch coverage), and Z be the repellent effect of ITNs. If $H_{(i,j)}$ is the number of hosts in patch (i,j), and $I_{(i,j)} = H_{(i,j)} Pc_{(i,j)} Z$ is the number of protected hosts in patch (i,j), then the number of unprotected hosts $(U_{(i,j)})$ in that particular patch is given by

$$U_{(i,j)} = H_{(i,j)} - I_{(i,j)} = H_{(i,j)} \left(1 - Pc_{(i,j)} Z\right). \tag{6}$$

If the patch does not have ITNs ($Pc_{(i,j)} = 0$), then $U_{(i,j)} = H_{(i,j)}$.

Since the repellent effect of ITNs affects host seeking mosquitoes, their dispersal rate into patches containing ITNs is affected. This effect is included by assuming that ITN repellency reduces hosts availability to mosquitoes in a given patch so that attractiveness of the patch to hosts seeking mosquitoes is reduced. Hosts covered by ITNs are therefore protected as some mosquitoes are repelled during the host seeking process. The dispersal rate, $\beta^H_{\xi'/(i,j)}$, detailed in [29] was modified by replacing the number of hosts present in a patch by those who are not protected by ITNs in the particular patch as:

$$\beta^H_{\xi'/(i,j)} = De^{-\lambda\left(\bar{U}^{ij}_{\xi'} - \bar{U}^{ij}_{\xi}\right)} \tag{7}$$

where $\bar{U}^{ij}_{\xi'}$ is the proportion of unprotected hosts available in patch ξ' contained in $c_{i,j}$ given by $\bar{U}^{ij}_{\xi'} = U^{\xi'} / H^{ij}_u$, and H_u is the total number of unprotected hosts in $c_{i,j}$. Here, $c_{i,j}$ is a set of seven patches sharing boundaries (patch (i,j) and its 6 neighbours). Simulations of the repellent effect are performed by considering that only unprotected hosts are attracting mosquitoes in each of the patches in the neighbourhood.

Spatial Clustering

Ecological models have been developed and used to study effects of landscape spatial heterogeneity on population dynamics [38–40] with increasing interest in the field of epidemiology [41]. Some models have been used to investigate spatial clustering effects in ecology [41–46]. To our knowledge, such methods have not been used by the malaria community to investigate clustering of vector control interventions. The degree of clustering (in the context of this study) is defined as a measure of the degree to which patches/hexagons on the hexagonal grid tend to spatially cluster together. In the context of vector control interventions, we define spatial clustering as a measure of the extent to which areas under interventions on a landscape are aggregated together. This degree varies from 0 (if the spatial distribution of interventions is random) to 1 (if the spatial distribution of interventions is highly concentrated on a certain portion of the landscape, or highly grouped together).

To evaluate the effect of spatial clustering of interventions using the model, we distributed interventions on the spatial grid [29]. The spatial distribution of interventions was varied according to the degree of spatial clustering chosen. These spatial clusters used for distributing interventions were created using the pair approximation method [38,39]. Two pair states were used: intervention and non-intervention states. These two states were assigned after defining a coverage area (that is proportion of patches assumed to be under interventions). Following Hiebeler [39], the degree of clustering, q_{00} was defined as the probability that a randomly chosen neighbour to a patch with intervention also contains the intervention. Spatial clusters of varying degrees on the model grid were created in Matlab using the steps detailed by Hiebeler [39]. Several configurations of spatial clusters were created from different initial random distributions of the intervention states to account for stochasticity of the method. Figure 2 illustrates one such cluster configuration produced at different degrees of clustering, q_{00}, when intervention coverage is 50% over the entire grid.

For the vector control investigations, cluster configurations were created at 10%, 30%, 50%, and 70% coverage levels, with the degree of spatial clustering, q_{00} ranging from 0 to 1 at an interval of 0.1. However, it is only possible to create spatial clusters when $q_{00} \geq 2 - (1/p_0)$ [39] (where p_0 represent intervention coverage). This was due to the fact that when an intervention coverage is high, it is likely that neighbours of patches under intervention, are also under intervention. This implies a lower bound on q_{00} for high coverage. For example, at $p_0 = 70\%$, the lower bound for $q_0 0$ is 0.57. This means that, it is not possible to create clusters at a degree of spatial clustering less than 0.57.

Model Parameterizations and Assumptions

Parameter values on stage specific mortality, and development rates used to simulate the model are given in Table 1. Various experimental studies show that ITN killing efficacy is variable [47,48] as it depends on local entomological and epidemiological conditions [49]. For the parameter values of interventions, we make the assumption that ITNs and IRS are 80% efficacious so that ε_{ITN} and ε_{IRS} were fixed at 0.8.

When a larvicide is applied to a breeding site, all larvae experience an increased mortality. Field studies show that larviciding is likely to kill all larvae when applied to a breeding site [33–35]. However, not all breeding sites can be identified for larvicidal treatment. Here, $80\% (\epsilon_{LV} = 0.8)$ of the breeding sites inside a patch are assumed to be identified and treated with larvicide. Thus, leaving 20% of breeding sites within a patch without larvicide, allowing larvae develop into pupae. We also

make the assumption that larvae are distributed uniformly across breeding sites.

Field studies on mark release recapture experiments of *Anopheles gambiae* also show that daily flight range from 200 to 400 m [50] or 800 m a day [9]. Others show that about 90% of mosquitoes reach a distance of 1.5 km. These experimental results indicate that mosquito flight distance is variable. Due to these variations,the total area modelled in this study was limited to one square kilometre. The patch size, with patch centroids 50 m apart and used in this work, was based on flight distances of mosquitoes chosen and numerical ease.

A 25 by 21 hexagonal grid was used as a hypothetical representation of a landscape. At the edges of the grid, periodic boundary conditions were used. This assumes the area being modelled is comparable to its neighbourhood. For simplicity, simulations were performed with all hexagons (patches) on the grid containing breeding sites and hosts. The dispersal related parameters for host seeking (β^H) and oviposition site searching (β^B) mosquitoes depend on the availability of hosts and breeding sites respectively and the diffusion rate, $D = 0.2$ per time was used in all simulations. The diffusion coefficient of dispersal ($D^* = D/A$, where A is the area of each patch contained in the hexagonal grid) scales with patch size and as a result, the equilibrium results presented in this study scale with increasing patch size or increasing number of patches (and total area modelled).

Measuring Intervention Effectiveness

We define intervention effectiveness as the reduction in the total equilibrium population of host seeking mosquitoes, over all

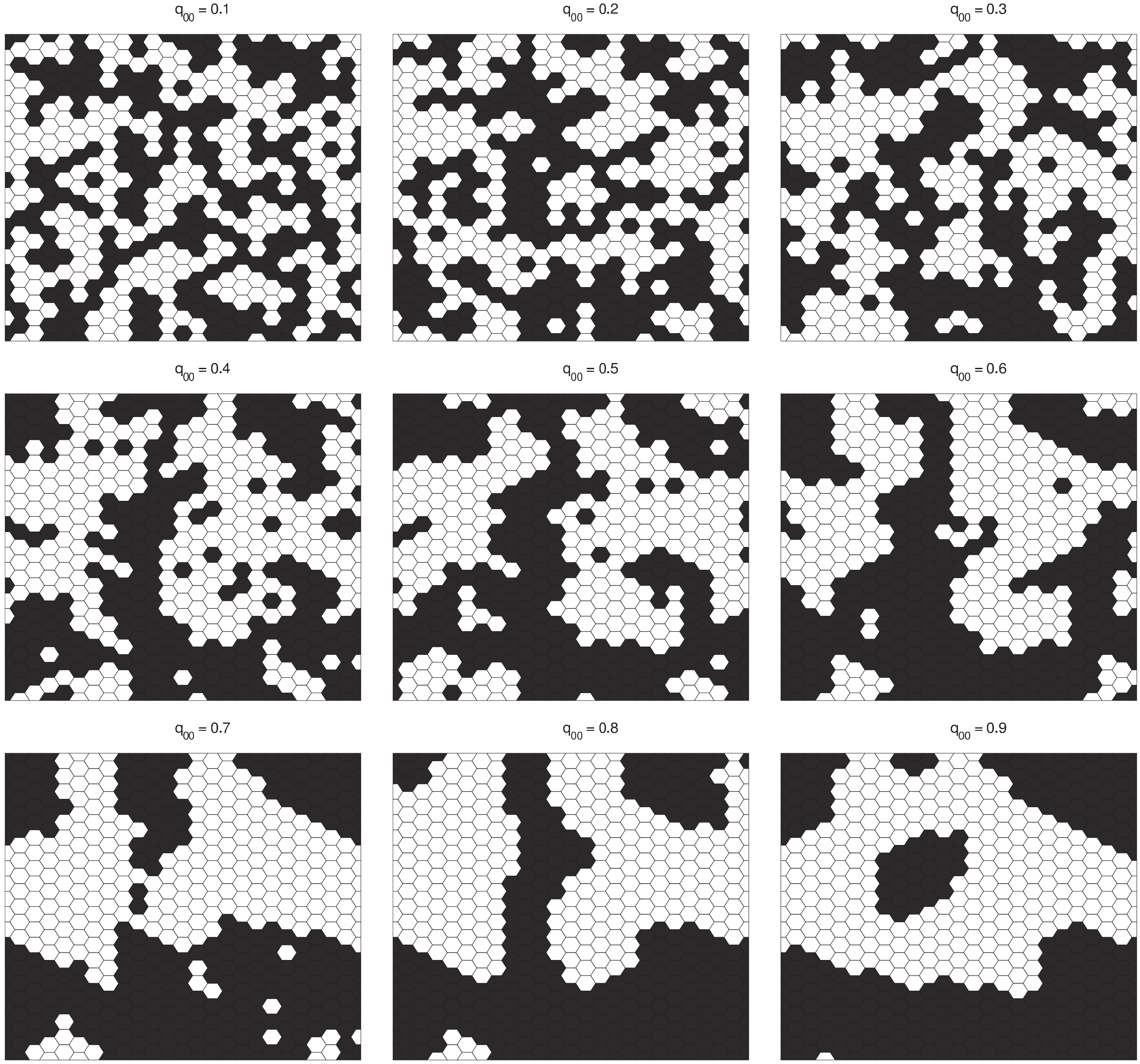

Figure 2. An example of spatial clusters generated at different degrees of clustering (q_{00}). An example of spatial clusters generated at different degrees of clustering (q_{00}) with a coverage of $p_0 = 0.5$ for the covered states (white) for intervention deployment and uncovered states (black). Clustering increases with increasing q_{00}.
doi:10.1371/journal.pone.0097065.g002

patches on the grid. In malaria transmission control, the number of potentially infective mosquitoes should be reduced. Thus, only host seeking adults, which transmit malaria, are considered. From the model, the equilibrium total number of host seeking mosquitoes is calculated over the entire grid as

$$A_h^* = \sum_{\xi \in \Xi} A_{h\xi}^*, \qquad (8)$$

where $A_{h\xi}$ is the equilibrium number of adult host seeking mosquitoes in patch ξ and Ξ is the set of all patches on the entire grid. In this context, we calculate intervention effectiveness, ϵ_{int}, as the proportionate reduction of an equilibrium population of host seeking mosquitoes, namely

$$\epsilon_{\text{int}} = 1 - \frac{A_h^{*(\text{int})}}{A_h^*}, \qquad (9)$$

where A_h^* is the equilibrium population of host seeking mosquitoes in the absence of interventions, and $A_h^{*(\text{int})}$ is the equilibrium population of host seeking mosquitoes in the presence of an intervention.

Simulations

Simulations were carried out in Matlab 7.10.0 (R2010a). The adaptive step size Runge-Kutta method of fourth and fifth order (*ode45*) was used to solve the system of ordinary differential equations (Eqn. (1)). Simulations were performed at intervention coverage levels of 0% coverage (no intervention), 10%, 30%, 50%, and 70%. The 0% level scenario was included to compute intervention effectiveness (Equation 9).

Several simulations were performed in this study. The first set of simulations involved creations of cluster configurations at each value of q_{00} as described in the spatial clustering subsection. A total of four cluster configurations were generated for each q_{00}. After clusters were generated, each cluster (a matrix of zeros and ones) for each q_{00} at each coverage level was used as an input matrix for placing interventions. Interventions were placed in entries with ones and entries with zeros represented non-intervention areas. One simulation was performed for each cluster configuration for each intervention package. Simulations were run until the system (1) was at equilibrium. The resulting equilibrium values were recorded and used to evaluate intervention effectiveness. For each cluster configuration at each coverage, one simulation was performed to obtain the equilibrium value which was used as a baseline for computing effectiveness as described above.

For each scenario a representative total population of 2700 eggs, 1900 larvae, 2000 pupae, 2400 host seeking mosquitoes, 1800 resting, and 1200 oviposition site searching mosquitoes were initially distributed across the grid. Parameter values used to simulate the model are given in Table 1. We numerically tested that there exists only one equilibrium point given different initial conditions for both the non-intervention and intervention scenarios.

Statistical Analysis of the Relationship between Intervention Spatial Clustering and Effectiveness

Simulation results for each coverage level were further analysed using statistical methods. The aim was to quantify the relationships between effectiveness and the degree of spatial clustering of an intervention. Since the effectiveness is measured as the proportionate reduction in host seeking mosquitoes, its range lies within 0 and 1. Thus, robust generalized linear models with a logit link [51] were used. The outcome variable in each model was the simulation results of effectiveness of an intervention package with the explanatory variable being the degree of spatial clustering at a given coverage level of that particular intervention package.

Results

The effectiveness of ITNs, IRS, and larviciding is related to the degree of spatial clustering of interventions and coverage levels (Figure 3). When the coverage of larviciding and IRS is 10% (Figure 3A), simulation results indicate that these interventions tend to be more effective when highly clustered compared to low clustering. However, the benefits of highly clustering IRS are not statistically significant (Table 2). At 30% coverage, high clustering of IRS appears to be no longer more effective than low clustering. For larviciding, at 30% spatial coverage level, larviciding is more effective when highly clustered compared to when lowly clustered. For ITNs distributed at low coverages of 10% to 30% (Figure 3A–B), the intervention is more effective with a low degree of spatial clustering compared to with a high degree of spatial clustering (ITN effectiveness is negatively correlated to the degree of spatial clustering).

At a moderate intervention coverage level of 50% (Figure 3C), effectiveness of IRS and larviciding decreases with increasing clustering and distributing ITNs randomly in a non-clustered way is more beneficial than in a clustered way. At an intervention coverage level of 70% (Figure 3D), distributing interventions widely and randomly in a non-clustered manner is more effective than clustering for any of the interventions.

When interventions are combined (Figure 4), effectiveness decreases with increasing degree of spatial clustering, implying more benefits when widely distributed in space. However, the combination of IRS and larviciding was not associated with the degree of spatial clustering when coverage was less then 30%.

Effectiveness of an intervention at zero clustering is highest for ITNs and lowest for larviciding (given our parameter values) when interventions are singly deployed (Table 2). Effectiveness at zero clustering is highest when all interventions are combined together, but the additional effect over ITNs alone is small. The combination of IRS and larviciding had the lowest effectiveness at zero clustering, irrespective of the coverage level.

At lower spatial coverage levels of single interventions, the difference in effectiveness between one intervention and another decreases with increasing value of the degree of spatial clustering. This gap (difference) remains almost constant at high coverage levels (Figure 3). For combined interventions and at all coverage levels, there is almost no difference in effectiveness for all combinations of interventions that included ITNs (Figure 4). The effectiveness of a combination of IRS and larviciding is consistently lower across all coverage levels. In addition, the difference in effectiveness between a combination of IRS and larviciding and other combinations is always high. However, at lower coverage levels, this difference decreased with increasing degree of spatial clustering (Figure 4A and B).

The scatter plots also show that there is variability in effectiveness. These variations increase with increasing clustering (Figures 3 and 4), especially at low to moderate coverage levels.

Discussion

In this study, an existing mathematical model of mosquito dispersal [29] was extended to include vector control interventions. In order to distribute interventions heterogeneously across the

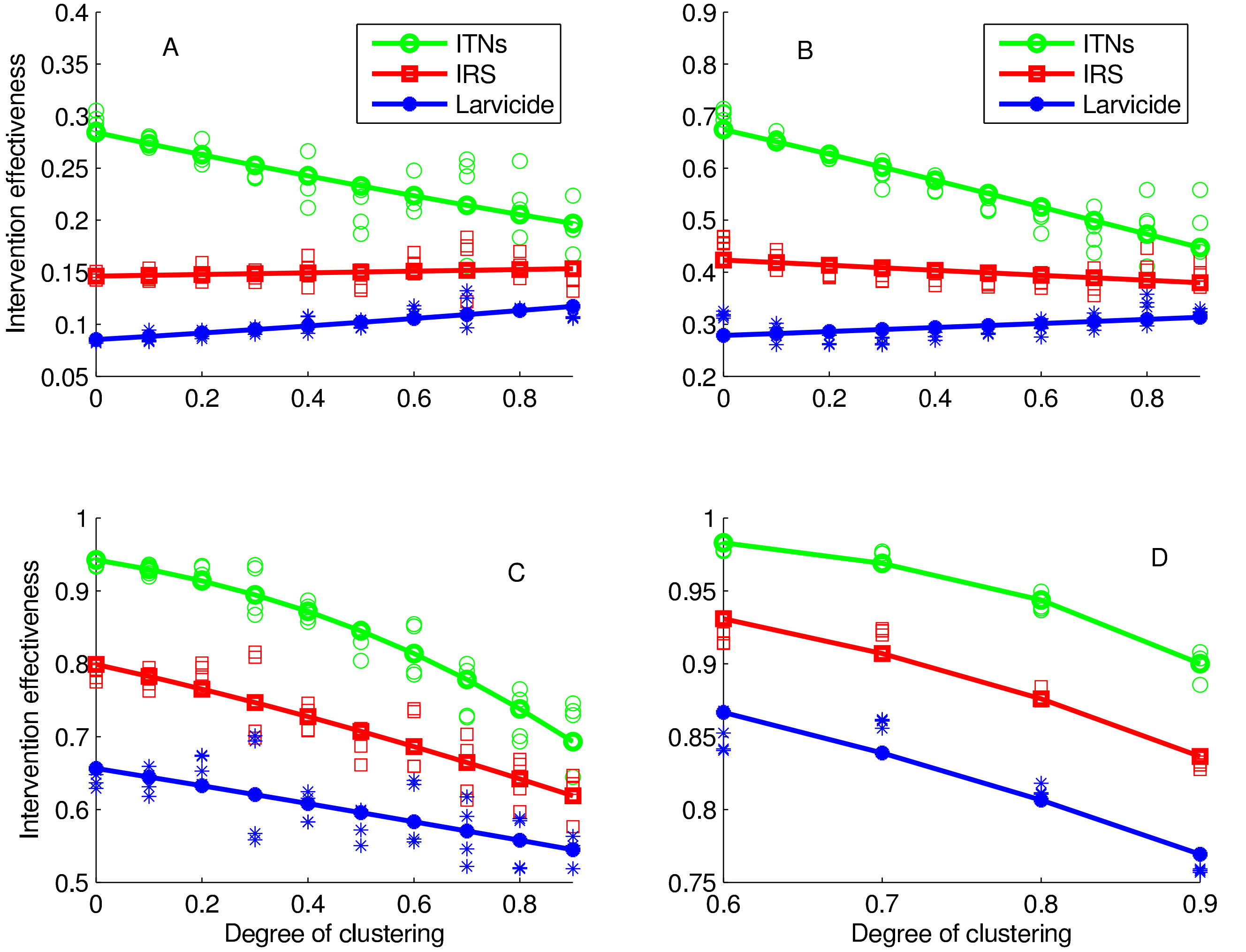

Figure 3. Intervention effectiveness by degree of spatial clustering of ITNs, IRS, and larviciding at different coverage levels. The symbols (scatter plots) represent simulated intervention effectiveness data from different configurations of intervention distribution to account for stochastic variations and the lines are the result of a linear fit on a logarithmic scale ($p=1/(1+\exp^{(-\beta_0-\beta_1 C)})$). Effectiveness is measured as the proportionate reduction of the equilibrium population of host seeking mosquitoes. Hosts and breeding sites were homogeneously distributed across the grid. Coverage levels A: 10%, B: 30%, C: 50%, and D: 70%.
doi:10.1371/journal.pone.0097065.g003

landscape, according to the degree of clustering chosen, this model was combined with an approach for modelling spatially heterogeneous landscapes [39] to assess the effects of spatial clustering of vector control interventions on their effectiveness, at various levels of spatial coverage and intervention combinations. As in another study [22], the reduction in the overall vector population density was used as an indicator of the population-wide effect of interventions. The results have important implications for deployment strategies in situations where universal coverage is not achievable.

Our model indicates that, with a single intervention of either IRS or larviciding in an environment where breeding sites and hosts are homogeneously distributed and spatial coverage of the intervention is low (i.e. few patches are covered), there is a small increase in effectiveness when deployment is highly spatially clustered compared to widely distributed in space. However, with high spatial coverage, it is more effective to distribute these interventions randomly in an unclustered manner. ITNs were less effective at a higher degree of clustering than at a lower degree of clustering for any spatial coverage level.

At a spatial coverage of less than 50%, if larviciding is highly clustered, then treated areas become almost mosquito free. However, if larviciding is not clustered, mosquitoes that breed in neighbouring patches can still feed in areas that have been larvicided. If coverage is moderate to high (50% or larger), larviciding is more effective when randomly distributed and unclustered, because a greater proportion of the remaining adult mosquitoes is likely to encounter the intervention when ovipositing. When larviciding is clustered, most of the ovipositing occurs in non-larvicided areas because adult mosquitoes are rare in larvicided areas. When larviciding is widespread and unclustered, a proportion of adult mosquitoes emerging in non-larvicided patches will migrate to, feed and oviposit in larvicided breeding sites.

With adulticidal interventions, especially ITNs, the benefits of distributing the intervention widely and unclustered are greater, because the mosquitoes need to avoid intervention patches each gonotrophic cycle if they are to survive. Where adulticidal interventions are clustered, mosquitoes emerging in locations remote from the intervention area are unlikely to be killed,

Table 2. Association between intervention effectiveness and the degree of spatial clustering of interventions by coverage level.

Coverage	10%	30%	50%	70%
Effectiveness at zero clustering (β_0) (logit transformed)[a]				
ITNs	−0.92 (0.02)	0.73 (0.04)	2.80 (0.06)	6.56 (0.41)
IRS	−1.77(0.02)	−0.31 (0.03)	1.38 (0.04)	3.49 (0.28)
Larvicide	−2.37 (0.02)	−0.95 (0.04)	0.65 (0.04)	2.29 (0.24)
All	−0.82 (0.02)	0.88 (0.04)	3.14 (0.07)	7.78 (0.46)
ITNs and IRS	−0.86 (0.02)	0.82 (0.04)	3.01 (0.07)	7.41 (0.45)
ITNs and larviciding	−0.87 (0.02)	0.82 (0.04)	3.03 (0.07)	7.51 (0.45)
IRS and larviciding	−1.55 (0.02)	0.00 (0.04)	1.93 (0.05)	6.21 (0.38)
Effect of clustering (β_1) on the effectiveness (logit scale)				
ITNs	−0.54 (0.07)	−1.04 (0.10)	−2.20 (0.12)	−4.75 (0.50)
IRS	0.06 (0.05)[b,c]	−0.20 (0.07)	−0.99 (0.07)	−1.95 (0.36)
Larviciding	0.39 (0.04)[b]	0.19 (0.07)[b]	−0.52 (0.06)	−1.09 (0.32)
All	−0.61 (0.07)	−1.17 (0.10)	−2.5(0.14)	−6.02 (0.55)
ITNs and IRS	−0.59 (0.07)	−1.11 (0.10)	−2.42 (0.13)	−5.64 (0.54)
ITNs and larviciding	−0.57 (0.07)	−1.11 (0.10)	−2.44(0.13)	−5.75 (0.54)
IRS and larviciding	−0.05 (0.05)[c]	−0.43 (0.08)[c]	−1.46 (0.09)	−4.73 (0.49)

Association between intervention effectiveness and the degree of spatial clustering of interventions by coverage levels. β_1 is an estimate (gradient) of the effect of the degree of spatial clustering of an intervention and β_0 is an intercept measuring the effectiveness of the intervention at zero clustering. The higher β_0, the higher the effectiveness at zero clustering. Figures in parenthesis are standard errors.

[a]$\beta_0 = \ln\left(\frac{p_0}{1-p_0}\right)$, where p_0 is the actual effectiveness.

[b]Positive relationship, implying a benefit of clustering the intervention.

[c]Not statistically significant (i.e. p-value >0.05).

doi:10.1371/journal.pone.0097065.t002

whereas when interventions are non-clustered, a mosquito will encounter them sooner or later. Consequently, at any spatial coverage level, average biting densities are reduced more by deploying ITNs in an unclustered manner than by clustering them. It also follows that widespread distribution of adulticidal interventions will reduce the number of old (potentially disease-transmitting) mosquitoes even more than it will reduce average densities. This finding, that the overall effect in the reduction of mosquito numbers is much greater if the intervention is spatially non-clustered and widely distributed, especially when coverage is moderate and insufficient to achieve universal coverage, contradicts the notion that a locally high coverage is needed to achieve a mass effect of ITNs or IRS for reduction in disease.

Highly clustered scenarios had lower ITN effectiveness. This is likely due to the fact that when intervention coverage is high, then the likelihood that any patch and its six neighbours are under intervention is high. In this aspect, patch attractiveness to biting mosquitoes is reduced. When this occurs, then all neighbouring patches produce the same repellency effect which results into fewer mosquitoes leaving the centre patch (because they are also repelled for each of their neighbours). In so doing, the repellency effect decreases and the killing effect becomes the main factor, rather than the combination of both repellency and killing.

While non-clustered deployment of most intervention packages is generally most effective, this may be expensive to achieve since it requires delivery even to remote locations. Interventions are often delivered preferentially to more accessible areas, and such clustered (and sometimes inequitable) distributions are likely to be the cheapest. To investigate how delivery costs affect cost-effectiveness, there is a need for modelling of different distribution schemes (for example for ITNs or IRS) of interventions given a fixed budget in various settings with different degrees of clustering, coverage levels and accessibility.

Efficacy, defined as the effect on the target stage of the vector as a proportion of the theoretical maximum effect, translates differently into effectiveness defined on some common metric of levels of transmission, disease control, or, in this paper, densities of host seeking mosquitoes. We have assumed 80% efficacious interventions throughout, and our results are consistent with other modelling work suggesting that at constant efficacy, ITNs have the highest impact on biting densities of mosquitoes [16,22] and in our simulations any combination of interventions which includes ITNs is also highly effective at all levels of coverage and across all spatial clustering. This may be accounted by the repellency effect of ITNs included in the model. The assumed 80% efficacy of ITNs in this work is representative of both the killing and repellency action of ITNs and of indoor biting coverage of individuals within a patch. Even with small patch sizes assuming an 80% efficacy for ITNs is likely too high. A further extension of the models would be to vary the level of intervention within each patch, and thus efficacy.

Comparing of Figures 3 and 4 indicates that although ITNs provide better protection alone compared to other interventions, results show that there are additional benefits if ITNs are combined with other interventions. Our study also shows that although larviciding is less effective compared to ITNs and IRS, treating a similar or higher level of coverage would result in a higher reduction of biting mosquitoes.

The current results are indicative of the effect of applying interventions within a small village, with a small number of dwellings or breeding sites per patch, but should also be broadly applicable to smaller patches corresponding to single individuals or breeding sites. We would not necessarily expect the same results to hold with very large patches, e.g. corresponding to whole villages where patch size might be comparable to the flight range of the mosquitoes and where other factors such as spatial variation within patches might be relevant.

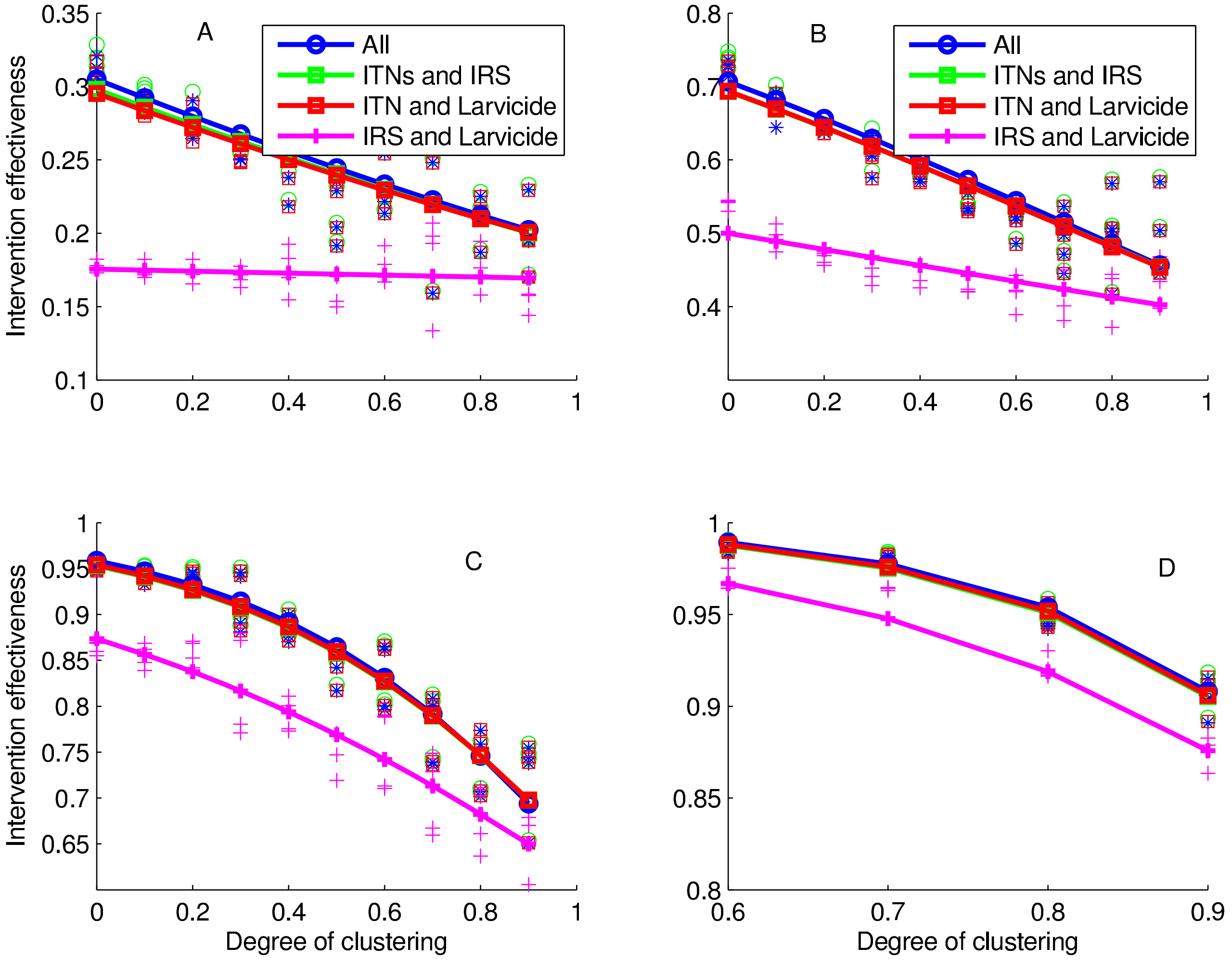

Figure 4. Effect of spatial clustering of interventions by coverage level for combined interventions. The symbols (scatter plots) represent simulated intervention effectiveness from different realizations depicting initial distribution of interventions before the process of clustering was undertaken to account for stochastic variations and the lines are the result of a linear fit on a logarithmic scale ($p = 1/(1+\exp^{(-\beta_0-\beta_1 C)})$). Hosts and breeding sites were homogeneously distributed over the grid. Coverage levels A: 10%, B: 30%, C: 50%, and D: 70%.
doi:10.1371/journal.pone.0097065.g004

Modelling and simulation provides a much easier approach to investigate these issues than field studies do, but inevitably require making simplifying assumptions. To assess the effect of clustering, we simulated a homogeneous distributions of both human hosts and breeding sites. The cues that these human hosts and breeding sites provide that influence movement of mosquitoes cancel each other out, therefore movement was not influenced by the availability of these hosts or breeding sites [52]. Further investigations need to incorporate scenarios in which breeding sites and hosts are heterogeneously distributed. In such scenarios, knowledge about hotspots will allow targeted (and therefore likely spatially clustered) deployment of interventions and this may well be more cost-effective than non-clustered deployment. In other words, in scenarios with spatially heterogeneous hosts and/or breeding sites, the cost of knowledge about where these are may well compensate for potential gains in effectiveness. However, in the absence of knowledge about spatial location of hosts and breeding sites for mosquitoes (even for scenarios when they are heterogeneously distributed) non-clustered distribution may be most cost-effective.

Results from this study provide evidence that the effectiveness of an intervention can be highly dependent on its spatial distribution. Given logistical and financial constraints, vector control plans should consider the spatial arrangement of any intervention package to ensure effectiveness is maximized. In the case of high achievable coverage, and in the absence of information that allows targeting, it is of great help to ensure that the distribution is as equitable and as evenly spatially spread as possible for maximizing benefits.

Acknowledgments

We thank our colleagues in the malaria modelling group at the Swiss TPH for their contributions and critical comments they provided. We also thank the reviewers for their critical review of this manuscript. The author thank the Swiss Tropical and Public Health Institute and the Ifakara Health Institute for their support.

Author Contributions

Conceived and designed the experiments: AML. Performed the experiments: AML. Analyzed the data: AML. Contributed reagents/materials/analysis tools: AML MAP TAS NC OB. Wrote the paper: AML. Provided critical input on designing the experiments: MAP TAS NC OB. Provided critical input on the experiments: MAP. Provided critical input on data analysis: MAP TAS NC OB. Provided critical input on writing the manuscript: TAS MAP NC OB.

References

1. WHO (2007) Insecticide treated mosquito nets: a WHO position statement. Technical report, World Health Organization.
2. Bayoh MN, Mathias DK, Odiere MR, Mutuku FM, Kamau L, et al. (2010) *Anopheles gambiae*: historical population decline associated with regional distribution of insecticide-treated bed nets in western Nyanza province, Kenya. Malaria Journal 9.
3. Kroeger A, Horstick O, Riedl C, Kaiser A, Becker N (1995) The potential for malaria control with the biological larvicide Bacillus thuringiensis israelensis (Bti) in Peru and Ecuador. Acta Tropica 60.
4. Lengeler C (2004) Insecticide-treated bed nets and curtains for preventing malaria. Cochrane Database of Systematic Reviews.
5. Muturi EJ, Burgess P, Novak RJ (2008) Malaria vector management: where have we come from and where are we headed? The American Journal of Tropical Medicine and Hygiene 78.
6. WHO (2004) Global strategic framework for integrated vector management. Technical report, World Health Organization, Geneva.
7. WHO (2011) World Malaria Report. Technical report, World Health Organization, Geneva.
8. RBM (2013) Minutes of roll back malaria vector control working group 8th annual meeting. Technical report, Roll Back Malaria. Available: http://www.rollbackmalaria.org/mechanisms/vcwg.html.
9. Gillies MT (1961) Studies on the dispersion and survival of *Anopheles Gambiae Giles* in East Africa, by means of marking and release experiments. Bulletin of Entomological Research 52: 99–127.
10. Gillies MT, Wilkes TJ (1978) The effect of high fences on the dispersal of some West African mosquitoes (Diptera: Culicidae). Bulletin of Entomological Research 68: 401–408.
11. Gillies MT, Wilkes TJ (1981) Field experiments with a wind tunnel on the flight speed of some West African mosquitoes (Diptera: Culicidae). Bulletin of Entomological Research 71: 65–70.
12. Service MW (1997) Mosquito dispersal - The long and the short of it. Journal of medical entomology 34.
13. Edman J, Scott T, Costero A, Morrison A, Harrington L, et al. (1998) *Aedes aegypti* (diptera culicidae) movement influenced by availability of oviposition sites. Journal of Medical Entomology 35: 578–583.
14. Cummins B, Cortez R, Foppa IM, Walbeck J, Hyman JM (2012) A Spatial Model of Mosquito Host-Seeking Behavior. PLoS Computational Biology 8.
15. McKenzie FE, Samba EM (2004) The role of mathematical modeling in evidence-based malaria control. The American Journal of Tropical Medicine and Hygiene 71: 94–96.
16. Chitnis N, Schapira A, Smith T, Steketee R (2010) Comparing the effectiveness of malaria vectorcontrol interventions through a mathematical model. The American Journal of Tropical Medicine and Hygiene 83.
17. Eckhoff PA (2011) A malaria transmission-directed model of mosquito life cycle and ecology. Malaria Journal 303: 10.
18. Griffin JT, Hollingsworth TD, Okell LC, Churcher TS, White M, et al. (2010) Reducing *Plasmodium falciparum* malaria transmission in Africa: A model-based evaluation of intervention strategies. PLoS Medicine 7.
19. Gu W, Novak RJ (2009) Agent-based modelling of mosquito foraging behaviour for malaria control. Transactions of the Royal Society of Tropical Medicine and Hygiene 103: 1105–1112.
20. Gu W, Regens JL, Beier JC, Novak RJ (2006) Source reduction of mosquito larval habitats has unexpected consequences on malaria transmission. PNAS 103.
21. Menach LA, Takala S, McKenzie FE, Andre P (2007) An elaborated feeding cycle model for reductions in vectorial capacity of night-biting mosquitoes by insecticide-treated nets. Malaria Journal 6: 10.
22. White MT, Griffin JT, Churcher TS, Ferguson NM, Basanez MG, et al. (2011) Modelling the impact of vector control interventions on *Anopheles gambiae* population dynamics. Parasites and Vectors 4.
23. Worrall E, Connor SJ, Thomson MC (2007) A model to simulate the impact of timing, coverage and transmission intensity on the effectiveness of indoor residual spraying (IRS) for malaria control. Tropical Medicine and International Health 12.
24. Yakob L, Yan G (2009) Modeling the effects of integrating larval habitat source reduction and insecticide treated nets for malaria control. PLoS ONE 4.
25. Yakob L, Yan G (2010) A network population model of the dynamics and control of African malaria vectors. Transactions of the Royal Society of Tropical Medicine and Hygiene 104: 669–675.
26. Gu W, Novak RJ (2009) Predicting the impact of insecticide-treated bed nets on malaria transmission: the devil is in the detail. Malaria Journal 256: 8.
27. Nourridine S, Teboh-Ewungkem MI, Ngwa GA (2011) A mathematical model of the population dynamics of disease - transmitting vectors with spatial consideration. Journal of Biological dynamics 5: 335–365.
28. Otero M, Schweigmann N, Solari HG (2008) A Stochastic Spatial Dynamical Model for *Aedes Aegypti*. Bulletin of Mathematical Biology 70: 1297–325.
29. Lutambi AM, Penny MA, Smith T, Chitnis N (2013) Mathematical modelling of mosquito dispersal in a heterogeneous environment. Mathematical Biosciences 241: 198–216.
30. Takken W (2002) Do insecticide-treated bednets have an effect on malaria vectors? Tropical Medicine and International Health 7.
31. Hawley WA, Phillips-Howard PA, Ter Kuile FO, Terlouw DJ, Vulule JM, et al. (2003) Communitywide effects of permethrin-treated bed nets on child mortality and malaria morbidity in western Kenya. The American Journal of Tropical Medicine and Hygiene 68: 121–7.
32. WHO (2006) Indoor residual spraying: Use of indoor residual spraying for scaling up global malaria control and elimination. Technical report, World Health Organization.
33. Fillinger U, Lindsay SW (2006) Suppression of exposure to malaria vectors by an order of magnitude using microbial larvicides in rural Kenya. Tropical Medicine and International Health 11.
34. Majambere S, Lindsay SW, Green C, Kandeh B, Fillinger U (2007) Microbial larvicides for malaria control in The Gambia. Malaria Journal 6: 76.
35. Mwangangi JM, Kahindi SC, Kibe LW, Nzovu JG, Luethy P, et al. (2011) Wide-scale application of Bti/Bs biolarvicide in different aquatic habitat types in urban and peri-urban Malindi, Kenya. Parasitology Research 108: 1355–1363.
36. Majambere S, Pinder M, Fillinger U, Ameh D, Conway DJ, et al. (2010) Is mosquito larval source management appropriate for reducing malaria in areas of extensive flooding in The Gambia? A cross-over intervention trial. The American Journal of Tropical Medicine and Hygiene 82.
37. Killeen GF, Tanner M, Mukabana WR, Kalongolela MS, Kannady K, et al. (2006) Habitat targeting for controlling aquatic stages of malaria vectors in Africa. The American Journal of Tropical Medicine and Hygiene 74.
38. Hiebeler D (1997) Stochastic spatial models: From simulations to mean field and local structure approximations. Journal of Theoretical Biology 187.
39. Hiebeler D (2000) Populations on fragmented landscapes with spatially structured heterogeneities: Landscape generation and local dispersal. Ecology 81.
40. Okuyama T (2008) Intraguild predation with spatially structured interactions. Basic and Applied Ecology 9: 135–144.
41. Hiebeler D (2005) A cellular automaton SIS epidemiological model with spatially clustered recoveries. In: Sunderam VS, van Albada GD, Sloot PMA, Dongarra J, editors, International Conference on Computational Science (2). Springer, number 3515 in Lecture Notes in Computer Science, 360–367.
42. Lee SH, Su NY, Bardunias P (2007) Exploring landscape structure effect on termite territory size using a model approach. BioSystems 90.
43. Su M, Li W, Li Z, Zhang F, Hui C (2009) The effect of landscape heterogeneity on host-parasite dynamics. Ecological Research 24: 889–896.
44. Thomson NA, Ellner SP (2003) Pair-edge approximation for heterogeneous lattice population models. Theoretical Population Biology 64: 271–280.
45. Tsonis AA, Swanson KL, Wang G (2008) Estimating the clustering coefficient in scale-free networks on lattices with local spatial correlation structure. Physica A 387.
46. Westerberg L, Ostman O, Wennergren U (2005) Movement effects on equilibrium distributions of habitat generalists in heterogeneous landscapes. Ecological Modelling 188: 432–447.
47. Chouaibou M, Simard F, Chandre F, Etang J, Darriet F, et al. (2006) Efficacy of bifenthrinimpregnated bednets against *Anopheles funestus* and pyrethroid-resistant *Anopheles gambiae* in North Cameroon. Malaria Journal 5: 77.
48. Oxborough RM, Mosha FW, Matowo J, Mndeme R, Feston E, et al. (2008) Mosquitoes and bednets: testing the spatial positioning of insecticide on nets and the rationale behind combination insecticide treatments. Annals of Tropical Medicine and Parasitology 102.
49. Smith DL, Hay SI, Noor AM, Snow RW (2009) Predicting changing malaria risk after expanded insecticide-treated net coverage in Africa. Trends in Parasitology 25.
50. Midega JT, Mbogo CM, Mwambi H, Wilson MD, Ojwang G, et al. (2007) Estimating dispersal and survival of *Anopheles gambiae* and *Anopheles funestus* along the kenyan coast by using markrelease-recapture methods. Journal of Medical Entomology 44: 923–929.
51. Papke LE, Wooldridge MJ (1996) Econometric methods for fractional response variables with an application to 401(K) plan participation rates. Journal of applied econometrics 11: 619–632.
52. Killeen GF, McKenzie FE, Foy BD, Bogh C, Beier JC (2001) The availability of potential hosts as a determinant of feeding behaviours and malaria transmission by African mosquito populations. Transactions of the Royal Society of Tropical Medicine and Hygiene 95.
53. Service MW (2004) Medical Entomology for Students. Cambridge University Press, Third edition.

54. Holsetein MH (1954) Biology of *Anopheles Gambiae*: research in French West Africa. Monograph series number 9, World Health Organization, Palais des Nations, Geneva.
55. Yaro AS, Dao A, Adamou A, Crawford JE, Ribeiro JMC, et al. (2006) The distribution of hatching time in *Anopheles gambiae*. Malaria Journal 5: 19.
56. Bayoh MN, Lindsay SW (2003) Effect of Temperature on the development of the aquatic stages of *Anopheles gambiae sensu stricto* (Diptera: Culicidae). Bulletin of Entomological Research 93: 375–381.
57. Kirby MJ, Lindsay SW (2009) Effect of temperature and inter-specific competition on the development and survival of *Anopheles gambiae sensu stricto* and *An. arabiensis* larvae. Acta Tropica 109: 118–123.
58. Gething PW, Van Boeckel TP, Smith DL, Guerra CA, Patil AP, et al. (2011) Modelling the global constraints of temperature on transmission of *Plasmodium falciparum* and *P. vivax*. Parasites and Vectors 92: 2.
59. Okogun G (2005) Life-table analysis of *Anopheles* malaria vectors: generational mortality as tool in mosquito vector abundance and control studies. Journal of Vector borne Diseases 42: 45–53.
60. Chitnis N, Smith T, Steketee R (2008) A mathematical model for the dynamics of malaria in mosquitoes feeding on a heterogeneous host population. Journal of Biological Dynamics 2: 259–285.
